# Use of serology to assess the probability of public health action needed for trachoma in coastal Ecuador

**DOI:** 10.64898/2026.02.18.26346552

**Published:** 2026-02-19

**Authors:** Everlyn Kamau, Lesly Simbaña Vivanco, Stuart Torres Ayala, Nikolina Walas, Gretchen Cooley, Chabier Coleman, E. Brook Goodhew, Diana L. Martin, Hadley Burroughs, Manuel Calvopiña, William Cevallos, Sandra Vivero, Victoria Nipaz, Josefina Coloma, Gwenyth O. Lee, Gabriel Trueba, Joseph N.S. Eisenberg, Karen Levy, Benjamin F. Arnold

**Affiliations:** 1. Francis I. Proctor Foundation, University of California, San Francisco, CA, USA; 2. Department of Microbiology, Universidad de San Francisco de Quito, Quito, Ecuador; 3. Division of Parasitic Diseases and Malaria, United States Centers for Disease Control and Prevention, Atlanta, GA, USA; 4. Universidad de las Americas, Facultad de Medicina, Carrera de Medicina, Quito, Ecuador; 5. Universidad Central del Ecuador, Instituto de Biomedicina, Quito, Ecuador; 6. Division of Infectious Diseases and Vaccinology, School of Public Health, University of California, Berkeley, CA, USA; 7. Rutgers Global Health Institute and Department of Biostatistics and Epidemiology, School of Public Health, Rutgers University, New Brunswick, NJ, USA; 8. Department of Epidemiology, University of Michigan, Ann Arbor, MI, USA; 9. Department of Environmental and Occupational Health Sciences, University of Washington, Seattle, WA, USA; 10. Department of Ophthalmology, University of California, San Francisco, CA, USA; 11. Institute for Global Health Sciences, University of California, San Francisco, CA, USA

## Abstract

We evaluated the probability of need for public health action against trachoma in Esmeraldas province, Ecuador. Compared to global references, seroconversion rates to *Chlamydia trachomatis* Pgp3 in children suggest high probability of action needed in rural villages (91%) and lower in more urban areas (32%), motivating further trachoma assessment.

## Background

Trachoma, a blinding disease caused by infection with ocular strains of *Chlamydia trachomatis (C. trachomatis)*, is targeted for elimination as a public health problem by the World Health Organization (WHO) defined as trachomatous inflammation–follicular (TF) in ages 1–9 years being <5% in each formerly endemic district, trachomatous trichiasis (TT) unknown to the health system among ages ≥15 years being <0.2% in each formerly endemic district and there being a system in place to manage TT [1]. Public-health interventions for trachoma include surgery for trichiasis; antibiotic mass drug administration (MDA); and programs to support facial cleanliness and environmental improvement (SAFE) [2].

*C. trachomatis* anti-Pgp3 IgG antibodies are a sensitive marker of past infection, making Pgp3 suitable for monitoring populations post-antibiotic MDA [3]. When measured in population-based surveys, Pgp3 can be translated into measures of transmission intensity [4], and a global analysis developed methods to estimate the probability of the need for public health action based on the Pgp3 seroconversion rate (SCR) [5].

The highest prevalence of trachoma is found in sub-Saharan Africa [2]. Trachoma remains a public health concern in vulnerable, remote areas in South America, notably Brazil, Colombia, Peru, and potentially Bolivia, Ecuador, and Venezuela, with ongoing Pan American Health Organization (PAHO)-supported efforts using the SAFE strategy [6]. Little is known about trachoma in Ecuador. We recently identified high levels of incident seroconversion to Pgp3 in a birth cohort in Esmeraldas Province in coastal Ecuador [7]. Here, we augment birth cohort samples to include older children from villages in the same region to enable estimates of the Pgp3 SCR and compare them with global distributions to estimate the probability of need for public health action against trachoma.

## Methods

### Study populations.

We included samples from two studies. The ECoMiD longitudinal birth cohort in Esmeraldas Province, which enrolled pregnant mothers from 2019 through 2022 along an urban-rural gradient that included Esmeraldas city (urban), Borbón (commercial center), four communities accessible by road, and four communities accessible via the Santiago and Onzole rivers (Supplementary Figure 1) [8]. Dried blood spots were collected between 2021 and 2024 when children were aged 6, 9, 12, 18, and 24 months [7]. We also included samples from community-based, annual serological surveillance for arbovirus transmission in Borbón plus five communities, four of which overlapped ECoMiD (Maldonado, Colon Eloy, Timbiré, Santo Domingo) plus an additional community, Santa Maria, on the Cayapas river [9]. Annual surveys were conducted between August to October and used stratified sampling, oversampling 2–14-year-olds. All serum samples collected from 2–15-year-olds in the 2021 and 2022 surveys were included in this analysis. Samples were tested for IgG to Pgp3 on the Luminex MAGPIX instrument at Universidad San Francisco de Quito [7].

We combined data from both cohorts and stratified the communities into two groups: Borbón (the commercial center for the region) and rural villages. We excluded birth cohort samples from Esmeraldas city (n=355) because the city was not included in the arbovirus surveillance study. Supplementary material includes details on study setting, sample testing and statistical analysis.

### Estimation of SCR and probability of public health action.

SCR is currently preferred to seroprevalence to guide decision-making [5]. We used SCR to assess probability of whether *C. trachomatis* transmission is present at a level that might warrant public health action. For each group, Borbon and rural villages, we fitted a catalytic model to the age-seroprevalence data to estimate a single seroconversion rate per 100 person-years using a generalized linear model (Supplementary Material), assuming a constant force of infection. Then given the SCR estimates, we estimated the probability of each group falling into either “Action needed” or “Action not needed” categories [5]:

P(Category|SCR)=P(SCR|Category)P(Category)

where “Action needed” are populations believed to likely experience development of disease sequelae and blindness from trachoma in the absence of effective interventions and “Action not needed” are populations believed unlikely to have sufficiently intense and sustained ocular *C. trachomatis* infections to lead to blindness, and thus no contemporary justification for population-level interventions.

The likelihood *P*(*SCR|Category*) was calculated as the empirical probability of the SCR estimates from Ecuador relative to SCRs in a global trachoma serology dataset derived from well characterized populations [10] (Supplementary Material). We considered the serological survey in coastal Ecuador a baseline survey (not necessarily post-elimination) and used an uninformative prior of category, i.e., *P*(*Category*) = 0.5, following previous analyses [5]. That is, since we identified no prior published information on trachoma epidemiology in coastal Ecuador, the communities were assumed equally likely to need trachoma-related public health action or intervention. For each group, we also calculated the probability that the SCR is above (or below) specific thresholds for elimination as a measure of confidence of the need for public health action. As no WHO guidelines on using SCR thresholds have yet been published, we considered SCR of 2 and 4 per 100 person-years as illustrative boundaries for evaluating trachoma elimination following previous analysis[5]. These values align with regions of high confidence (>90% probability) of “Action not needed” (SCR < 2) versus “Action needed” (SCR > 4) [5].

## Results

### Seroprevalence and seroconversion rates

**Figure 1.**
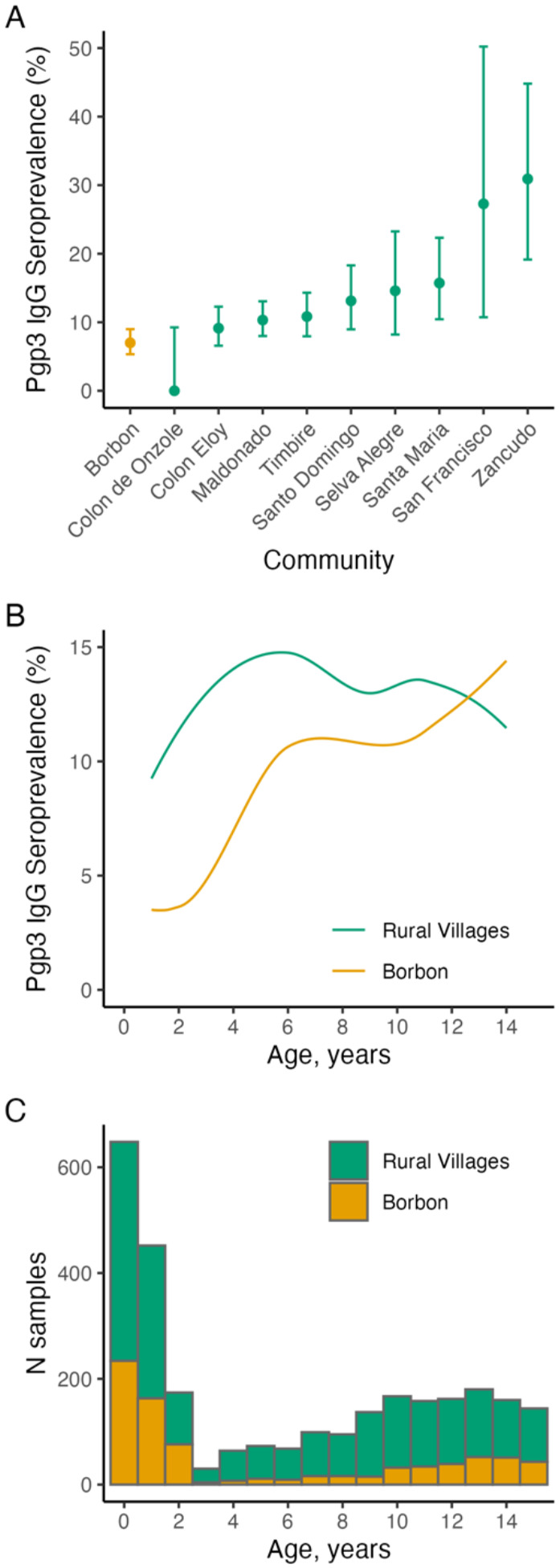
Seroprevalence to *Chlamydia trachomatis* Pgp3 in Esmeraldas province, Ecuador, 2021–2024. **A**. Seroprevalence among all children ages 0–15 years old in Borbon (commercial center) and in nine rural villages. **B.** Seroprevalence by age and community type estimated using locally weighted regression, trimmed to ages 1–14 years to reduce edge effects. **C**. Number of samples by age in years completed and community type. A total of 2,806 samples were collected from 1,243 children ages 0–15 years old. There were 790 samples from 401 children ages 1–5 years old.

A total of 2,806 dried blood spots from 1,243 children aged 0–15 years were analyzed for anti-Pgp3 IgG. Overall, seroprevalence was 7.0% (56/800, 95% CI 5.3% to 9.0%) in Borbon and ranged from 0% (0/38, 95% CI, 0% to 9.3%) in Colon de Onzole to 31% (17/55, 95% CI,19.1% to 44.8%) in Zancudo (Figure 1A). Age-specific seroprevalence rose earlier and to a higher level in rural villages compared with Borbón and groups converged by age 13 years (Figure 1B). Sample sizes were larger at the youngest ages through contributions from the birth cohort, with far fewer 3–5-year-olds contributing samples (Figure 1C).

Among 1–5-year-olds (401 children and 790 samples), the SCR was 4.6 (95% CI, 3.5 to 6.2) per 100 person-years. The SCR in 1–5-year-olds was 1.9 (95% CI, 0.7 to 5.5) per 100 person-years in Borbón and 5.7 (95% CI, 4.2 to 7.7) per 100 person-years in rural communities.

### Probability of need for public health action for trachoma

**Figure 2.**
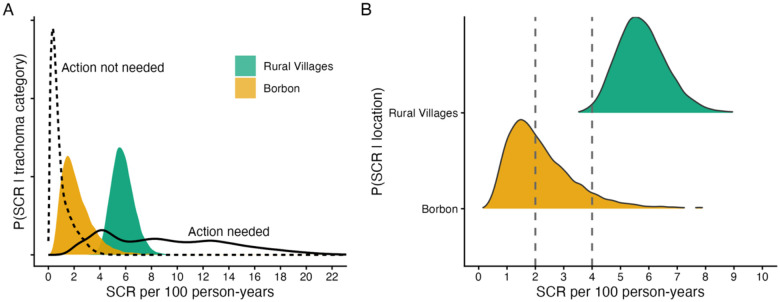
Model-based estimates of the *Chlamydia trachomatis* Pgp3 seroconversion rate (SCR) in Esmeraldas province, Ecuador compared to global trachoma serology distributions. **A.** Probability distributions of the SCR for Borbón (commercial center), and nine rural villages estimated among children 1–5 years old, super-imposed with pooled distributions of the SCR from populations classified as “Action not needed” (dashed line) and “Action needed” (solid line) regarding public health responses for trachoma control in a global analysis across a range of endemicity [5]. **B.** Probability distributions of the SCR for Borbón and rural villages from panel A compared with illustrative thresholds of 2 and 4 per 100 person-years. Below 2 has been identified as a region of >90% probability of public health Action not needed, whereas >4 is a region of ≥90% probability of public health Action needed.

When compared to distributions from 34 global trachoma surveys with expert determination of the need for public health action, the probability that public health action *is* needed based on the estimated SCR was 99.3% for the rural villages and 32.0% for Borbón (Figure 2A). Conversely, the probability that public health action *is not* needed (1 − *P*(*action is needed*)) was 0.7% in rural and 68% in Borbón. The probability that the SCR fell below 2 per 100 person-years (i.e., *P*(*SCR* ≤ 2), a region of high confidence for no public health action needed) was 0% for the rural villages and 52.4% for Borbón (Figure 2B). Conversely, the probability that SCR was above 4 (i.e., *P*(*SCR* ≥ 4)) was 91.3% for the rural villages and 8.8% for Borbón (Figure 2B).

## Discussion

Antibody responses from children in rural villages within the Santiago river basin suggest a high probability of public health action being needed for trachoma when compared to global reference distributions of Pgp3 SCR [10]. The SCR and seroprevalence estimates in rural villages fall within the range observed in districts with high levels of ongoing transmission requiring SAFE interventions [5], and are consistent with estimates in endemic districts considered to have persistent active trachoma in Ethiopia [11].

Efforts for trachoma elimination in the Americas, led by PAHO, have planned trachoma surveys in Ecuador, with priority surveillance in the Amazon basin [12]. Our data suggests that Esmeraldas Province should be considered as well. Although there are no formal WHO Pgp3 SCR thresholds for stopping or starting interventions, we used SCR of <2 and >4 as regions of high confidence for “Action not needed” versus “Action needed”. The results motivate additional trachoma surveillance (such as standardized baseline surveys, ophthalmologic assessments) and strengthened water, sanitation, and hygiene (WASH) interventions before initiating MDA in rural communities in the study communities. The results may warrant additional monitoring in Borbón, as the 95% CI of the SCR spans the intermediate range of 2–4 per 100 person-years.

These findings are relevant in the context of the Americas for identifying settings at risk of persistent or re-emerging transmission, particularly in rural areas characterized by limited access to safe WASH. Limitations of this analysis are that the sampling strategy did not use a population-based probability sample with many sampling clusters, as is commonly recommended for trachoma surveillance [13–15]’. One implication is that children aged 3–5 years were under-represented, particularly in Borbón. Additionally, we did not characterize blood samples with respect to clinical signs of trachoma or ocular *C. trachomatis* infection. A natural next step would be to conduct appropriate baseline surveys using standardized methodologies [13–15]’ to ascertain presence of active trachoma and provide reference points against which subsequent progress can be measured.

WHO aims to eliminate trachoma as a public health problem by 2030. This analysis provides a generalizable example of using existing serosurveys to inform public health decision-making as we approach elimination.

## Supplementary Material

1

## Data Availability

Replication files are available through the Open Science Framework (https://osf.io/najfz/). Due to the small population of the study region, UCSF IRB determined that individual-level data that included child age (essential for the analysis), even if de-identified, would be considered potentially identifiable. Please contact the corresponding author regarding possible access to data underlying this analysis, pending appropriate IRB approval.
