# Supplementary material for: Use of serology to assess the probability of public health action needed for trachoma in coastal Ecuador
Source: medRxiv. 2026 Feb 19:2026.02.18.26346552. Preprint. [Version 1] doi: 10.64898/2026.02.18.26346552 (PMC12934857; doi:10.64898/2026.02.18.26346552)
Supplement: 1 [file NIHPP2026.02.18.26346552V1-supplement-1.pdf]

medRxiv preprint doi: https://doi.org/10.64898/2026.02.18.26346552; this version posted February 19, 2026. The copyright holder for this preprint (which was not certified by peer review) is the author/funder, who has granted medRxiv a license to display the preprint in perpetuity.

It is made available under a CC-BY 4.0 International license .

SUPPLEMENTARY MATERIAL

Use of serology to assess the probability of public health action needed for trachoma in coastal Ecuador

Kamau et al.

Supplementary Methods

Study setting, sample testing and statistical analysis

ECoMiD is a longitudinal birth cohort study in Esmeraldas Province, on the northern coast of Ecuador [1]. The study enrolled women ages ≥18 years old in their third trimester (37 weeks' gestation) of pregnancy who were identified through prenatal care visits and outreach in local health clinics. The study excluded women with known, high-risk pregnancies, those who anticipated moving in the next 6 months, and those who planned to have a non-vaginal (caesarean) delivery. The study enrolled pregnant mothers from 2019 through 2022 along an urban-rural gradient Esmeraldas city (urban), Borbón (commercial center), four communities accessible by road, and four communities accessible via the Santiago and Onzole rivers (**Fig S1**). Children in the birth cohort were followed for their first two years of life, with biological samples collected every three months. The study region is predominantly lowland tropical rainforest and converted agricultural land that has undergone gradual development since road construction increased in the early 2000s, bringing increased connectivity between urban Esmeraldas city, the town of Borbón, and rural villages, which has led to changes in infectious disease transmission dynamics documented through the EcoDess research consortium [2].

Field staff collected dried blood spot samples from children up to five times at ages 6, 9, 12, 18, and 24 months. Dried blood spots were collected on filter paper, each with six 10 µL extensions. These were then dried, individually sealed with desiccant in plastic bags, and stored at –20°C in the field office before being shipped to the USFQ at ambient temperature and stored at -20°C.

Samples from children enrolled after Ecuador's COVID-19 pandemic shutdowns were the focus of IgG testing, spanning the 2021-2024 period. A single 10 µL extension from each participant's sample was eluted overnight at 4°C in a Buffer B(1X PBS pH 7.2-7.4, 0.5%casein, 0,5% PVA, 0.8% PVP, 0.3% Tween 20, 0.02%NaN3, 3 ug/ml of *E. coli* extract). Eluates were further diluted with Buffer B to a final concentration of 1:400 for use in the multiplex bead assay.

Fifty microliters of the eluted samples were incubated for 1.5 hours with microsphere beads with coupled antigens in a 45-plex assay with antigens to a broad set of enteric pathogens, vaccine

medRxiv preprint doi: https://doi.org/10.64898/2026.02.18.26346552; this version posted February 19, 2026. The copyright holder for this preprint (which was not certified by peer review) is the author/funder, who has granted medRxiv a license to display the preprint in perpetuity.

It is made available under a CC-BY 4.0 International license .

preventable diseases, arboviruses, and neglected tropical diseases, including Pgp3 and Ct694 (*C. trachomatis*).The concentration used was 1250 beads per well, diluted in assay buffer (1X PBS, 0.5% BSA, 0.05% Tween 20, 0.02% NaN3).

Samples were incubated for 45 minutes with a mixture of biotinylated mouse anti-human IgG (50ng/well) and IgG4 (40ng/well) from Southern Biotech, Birmingham, AL, USA. This was followed by a 30-minute incubation with R-phycoerythrin conjugated to streptavidin (250µg/well) from Invitrogen, Waltham, MA, USA, serving as a fluorescent marker. All incubations were performed at room temperature with agitation, with triple washes using PBST buffer (PBS with 0.05% Tween) between each step. Finally, the beads were resuspended in 100µL of PBS, and the plates were stored at 4°C until they were read the next day.

Bound IgG to each antigen was measured using median fluorescence intensity minus background (MFI-bg) on the Luminex platform using a MAGPIX instrument. Plates included Buffer B-only wells as blanks, along with a negative control and two pooled positive controls at different concentrations for quality assurance. Final MFI-bg values were obtained by subtracting the Buffer B blank from each antigen's MFI values. Half (11 of 22) plates were run in duplicate to quantify within- and between-plate variability in MFI-bg values. Plates were repeated if positive control monitoring targets fell outside two standard deviations of the mean in Levy-Jennings control chart.

Seropositivity cutoffs for Pgp3 (MFI=238) and Ct694 (MFI=710) were determined using external values from a receiver operator characteristic curve analysis of known positive and negative samples at the US CDC. We focused primarily on Pgp3 responses to align with elimination surveillance studies that rely primarily on Pgp3 and used Ct694 as an adjunctive measure [3,4].

We estimated seroprevalence as the proportion of samples positive with exact binomial (Clopper-Pearson) 95% confidence intervals. We combined data from both cohorts and stratified the communities into two groups: Borbón (the commercial center for the region) and rural villages. We excluded birth cohort samples from Esmeraldas city (n=355) because the city was not included in the arbovirus surveillance study. We estimated prevalence differences between the two groups (Borbón and rural villages) using a linear-binomial model with robust standard errors clustered at the child-level.

Estimation of SCR within a generalized linear model using maximum likelihood

The seroconversion rate (SCR) from a single-rate catalytic model assuming no seroreversion is closely tied to the slope of age-seroprevalence curve. The SCR is equal to the slope of the age-

medRxiv preprint doi: https://doi.org/10.64898/2026.02.18.26346552; this version posted February 19, 2026. The copyright holder for this preprint (which was not certified by peer review) is the author/funder, who has granted medRxiv a license to display the preprint in perpetuity.

It is made available under a CC-BY 4.0 International license .

seroprevalence curve divided by the complement of the seroprevalence at age A = a. It can be shown that the SCR based on this model can be estimated as the exponentiated intercept from a generalized linear model with binomial error structure and a complementary log-log link:

$$log[-\log(1 - P(Y = 1|A))] = log\lambda + logA$$

where Y represents individual-level serostatus (1: seropositive, 0: seronegative or equivocal), A is the child's age in years, and λ is the SCR. The SCR provides an estimate of the force of infection, an epidemiological parameter that denotes the rate at which susceptible individuals in the population become infected. In this analysis, we did not allow for seroreversion in the SCR estimation.

Estimation of probability of public health action – 'Action not needed' versus 'Action needed'

First, we downloaded posterior distributions of SCR estimates derived from a global trachoma serology dataset of well characterized populations (OSF (https://osf.io/ykjc4/) Dryad (https://datadryad.org/dataset/doi:10.5061/dryad.5qfttdzhx) [8]. These populations had been determined by a group of experts to either require further action (Action needed) or not (Action not needed) [3]. Therefore, the downloaded data could be labelled according to these two categories.

Then for the Ecuador data, we created a distribution of random values from the estimated log(SCR) and its standard error (SE):

$$\mathbb{R} = exp\big(rnorm(n, logSCR, logSE)\big) * 100$$

to create the likelihood that would be used in the calculation of probability as described below.

To calculate probability for each category of public health action, we used a mixture model framework applied to the distributions of SCR estimates. This approach assumes that an SCR estimate is drawn independently from a 2-component distribution of the two categories above, $k \in \{1, 2\}$. So, for each component or category (C_k: action not needed, action needed) and SCR estimate, $x \in \mathbb{R}$, we computed the posterior probability, $p(C_k|x)$, using Bayes' rule:

$$p(C_k|x) \propto p(C_k) * \frac{p(x|C_k)}{p(x)}$$

where $p(C_k)$ is the prior probability that a population is in category C_k. In this study, $p(C_k) = 0.5$ as we considered the Ecuador study as a baseline survey. $p(x|C_k)$, is the likelihood evaluated as empirical probability density function at each value x. In other words, the probability of x relative to the distribution of values comprising the category C_k.

medRxiv preprint doi: https://doi.org/10.64898/2026.02.18.26346552; this version posted February 19, 2026. The copyright holder for this preprint (which was not certified by peer review) is the author/funder, who has granted medRxiv a license to display the preprint in perpetuity.

It is made available under a CC-BY 4.0 International license .

$p(x)$ denotes the marginal likelihood or normalizing constant for the posterior density obtained by integrating the products of $p(x|C_k)$ and $p(C_k)$. That is, the sum of the products of the density function and prior probability for each k,

$$\sum_{k=1}^{2} \omega_k f_k(x|C_k)$$

or

$$p(x|C_{k=1})p(C_{k=1}) + p(x|C_{k=2})p(C_{k=2})$$

Estimating $\boldsymbol{P(SCR \leq c)}$

Using the simulated likelihood $\mathbb{R}$ above, we calculated $P(SCR \leq c)$ as a cumulative distribution function or the empirical probability density $\mathbb{R} \leq c$.

All analyses were done in R v4.5.0.

medRxiv preprint doi: https://doi.org/10.64898/2026.02.18.26346552; this version posted February 19, 2026. The copyright holder for this preprint (which was not certified by peer review) is the author/funder, who has granted medRxiv a license to display the preprint in perpetuity.

It is made available under a CC-BY 4.0 International license .

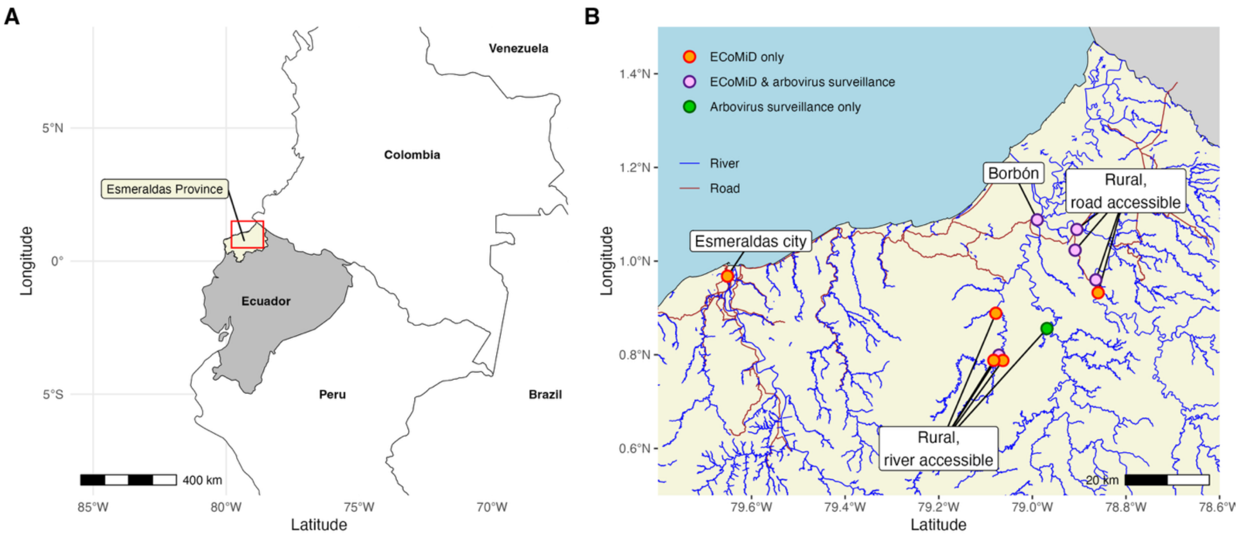

Supplementary Figure 1. Study location in Esmeraldas province, Ecuador. **A**. Esmeraldas province is in northern coastal, Ecuador, along the border with Colombia. **B.** Children were enrolled from Esmeraldas city (population approximately 162,000), the town of Borbón (population approximately 5,000), four road accessible rural villages (populations 500 to 1000) and five river-accessible rural villages (populations 200 to 700). This study focuses on samples from Borbon and rural villages. Major rivers (blue) and major roads (brown) provided by OpenStreetMap (https://www.openstreetmap.org).

medRxiv preprint doi: https://doi.org/10.64898/2026.02.18.26346552; this version posted February 19, 2026. The copyright holder for this preprint (which was not certified by peer review) is the author/funder, who has granted medRxiv a license to display the preprint in perpetuity.

It is made available under a CC-BY 4.0 International license .

Supplementary Material References

1. Lee, G. O. *et al.* Gut microbiome, enteric infections and child growth across a rural-urban gradient: protocol for the ECoMiD prospective cohort study. *BMJ Open* **11**, e046241 (2021).
2. Eisenberg, J. N. S. *et al.* Environmental change and infectious disease: how new roads affect the transmission of diarrheal pathogens in rural Ecuador. *Proc Natl Acad Sci U S A* **103**, 19460–19465 (2006).
3. Kamau, E. *et al.* Characterizing trachoma elimination using serology. *Nat Commun* **16**, 5545 (2025).
4. Tedijanto, C. *et al.* Monitoring transmission intensity of trachoma with serology. *Nat Commun* **14**, 3269 (2023).
5. Tedijanto, C. *et al.* Seroreversion to Chlamydia trachomatis Pgp3 Antigen Among Children in a Hyperendemic Region of Amhara, Ethiopia. *The Journal of Infectious Diseases* jiad602 (2023) doi:10.1093/infdis/jiad602.
6. West, S. K. *et al.* Longitudinal change in the serology of antibodies to Chlamydia trachomatis pgp3 in children residing in a trachoma area. *Sci Rep* **8**, 3520 (2018).
7. West, S. K., Munoz, B., Mkocha, H., Gaydos, C. A. & Quinn, T. C. The effect of Mass Drug Administration for trachoma on antibodies to Chlamydia trachomatis pgp3 in children. *Sci Rep* **10**, 15225 (2020).
8. Kamau, E. *et al.* Data Resource Profile: Global Trachoma Serology Data Repository. *International Journal of Epidemiology* **54**, dyaf183 (2025).
